# Burden of illness of trigeminal neuralgia among patients managed in a specialist center in England

**DOI:** 10.1186/s10194-020-01198-z

**Published:** 2020-11-10

**Authors:** Lasair O’Callaghan, Lysbeth Floden, Lisa Vinikoor-Imler, Tara Symonds, Kathryn Giblin, Chris Hartford, Joanna M. Zakrzewska

**Affiliations:** 1 Biogen, 225 Binney St, Cambridge, MA 02142 USA; 2 Clinical Outcomes Solutions, 1820 E. River Rd., Suite 220, Tucson, AZ 85718 USA; 3 Clinical Outcomes Solutions, Unit 68, Basepoint, Shearway Road, Shearway Business Park, Folkestone, Kent, CT19 4RH UK; 4 Formerly Biogen, 225 Binney St, Cambridge, MA 02142 USA; 5 Royal National ENT & Eastman Dental Hospitals, 4th Floor Central, 250 Euston Road, London, NW1 2PQ UK; 6 UCLH NHS Foundation Trust, Oral theme of the UCL/UCLH NIHR Biomedical Research Centre UK, London, UK

**Keywords:** Trigeminal neuralgia, Trigeminal nerve, Facial pain, Microvascular decompression, Multidisciplinary approach, Patient related outcomes

## Abstract

**Background:**

Trigeminal neuralgia (TN) causes severe episodic, unilateral facial pain and is initially treated with antiepileptic medications. For patients not responding or intolerant to medications, surgery is an option.

**Methods:**

In order to expand understanding of the pain-related burden of illness associated with TN, a cross-sectional survey was conducted of patients at a specialist center that utilizes a multidisciplinary care pathway. Participants provided information regarding their pain experience and treatment history, and completed several patient-reported outcome (PRO) measures.

**Results:**

Of 129 respondents, 69/128 (54%; 1 missing) reported no pain in the past 4 weeks. However, 84 (65%) respondents were on medications, including 49 (38%) on monotherapy and 35 (27%) on polytherapy. A proportion of patients had discontinued at least one medication in the past, mostly due to lack of efficacy (*n* = 62, 48%) and side effects (*n* = 51, 40%). A total of 52 (40%) patients had undergone surgery, of whom 30 had microvascular decompression (MVD). Although surgery, especially MVD, provided satisfactory pain control in many patients, 29% of post-surgical patients reported complications, 19% had pain worsen or stay the same, 48% were still taking pain medications for TN, and 33% reported new and different facial pain.

**Conclusions:**

In most PRO measures, respondents with current pain interference had poorer scores than those without pain interference. In the Patient Global Impression of Change, 79% expressed improvement since beginning of treatment at this clinic. These results indicate that while the multidisciplinary approach can substantially alleviate the impact of TN, there remains an unmet medical need for additional treatment options.

## Background

Trigeminal neuralgia (TN) is a rare condition that affects the trigeminal nerve, resulting in extreme, sporadic, sudden, electric shock-like unilateral facial pain [1]. The attacks typically last only for a few seconds to a maximum of 2 min and can occur in quick succession with a frequency of 1–50/day [2]. These episodes of TN, encompassing the duration of recurrent attacks, can last for periods of days to even months, but can go into periods of remission which can last for months [3]. The condition occurs most frequently in people over 50 years of age, and is more prevalent in women than in men [4]. The intensity and unpredictability of the pain can be physically and mentally incapacitating, and result in a severe burden of illness (BOI) and impaired patient quality of life (QoL) [5, 6]. Further, diagnostic delays [7], suboptimal management strategies, complications from treatments, and resistance to treatment may contribute to the disease burden [6].

Recently, TN was classified into 3 categories: classical (primary), in which vascular compression of the nerve with morphological changes in the trigeminal root is observed; secondary, in which major neurologic disease such as multiple sclerosis or tumor of the cerebellopontine angle has been identified; and idiopathic, in which no cause has been found [2, 8].

Primary treatment for TN has been pharmacological therapy, with the antiepileptic drugs (AEDs) carbamazepine and oxcarbazepine being first-line [8]; a range of other AEDs such as lamotrigine pregabalin, gabapentin, phenytoin, and baclofen are utilized when first-line drugs are ineffective or contraindicated [8]. However, large claims studies in the USA and smaller studies in Europe show frequent change of medication, suggesting poor efficacy and/or poor tolerability [9–11]. In addition, common side effects of these drugs in the TN population include significant cognitive impairment [12]. For patients non-responsive or intolerant to medications, surgery may be an option, with microvascular decompression (MVD) regarded as the most effective procedure [8]. Although neurosurgical procedures in selected TN patients can produce excellent results, it is not clear which patients will have good outcomes, as studies that have examined factors contributing to surgical outcomes have been limited [8].

There is considerable evidence that TN has a significant impact on QoL [5, 10, 13]. The natural history of TN has generally been considered progressive, with few remission periods and increasingly longer and more intense relapses gradually becoming less responsive to AEDs [14, 15]. However, recent studies suggest that patients managed in specialist centers utilizing a multidisciplinary approach may experience disease stabilization, and even improvement [5, 9, 10, 16, 17].

The current study is a cross-sectional component of a longitudinal survey project, and consists of assessments performed at a single timepoint, in a cohort of patients receiving a high level of clinical care at a specialist facility which has been managing TN patients over several years using a multidisciplinary care pathway. The aim of this study was to gain increased insight into the BOI of TN by looking at medication use, surgery, pain experience and quality of life as measured by several PROs, and to determine whether current treatments are meeting patient needs. The well-documented and consistently recorded medical and therapeutic history of these patients allowed a high level of specificity and accuracy in the analysis of the results.

## Methods

### Patient recruitment

Patients were recruited for the study in 2018 from a dedicated multidisciplinary facial pain clinic within a London teaching hospital. At this facility, if patients are taking medication(s), reviews are every 6 months on average; patients may be discharged if they are no longer taking medications following surgery. This single-site patient population has been managed longitudinally since 2007 in a specialized multidisciplinary team model, with access to physicians, dentists, clinical psychologists, a clinical nurse specialist, a neurologist and 3 neurosurgeons. The multidisciplinary team follows the hospital-approved guidelines, which are based on published guidelines for TN [18]. Patients at the facility are clinically contacted a minimum of twice yearly, either face to face, or by telephone by a clinical nurse specialist [17].

Patients were initially contacted and informed of the study by a member of the multidisciplinary team. Patients who were able to be contacted and were reported to have primary TN were invited to complete the survey. This was the first time they were being asked by the multidisciplinary facial pain clinic to complete a survey electronically.

### Survey methodology

This cross-sectional survey was deployed using a web-based system. If patients were unwilling or unable to complete the online form, they were offered a paper version of the survey. The survey included items about pain interference, history and experience of medication use, history and experience of surgery related to TN, and 5 PRO measures. The 5 PRO measures were: 1) The Penn-Facial Pain Scale-Revised (PENN-FPS-R), an instrument to assess patient-reported impact of facial pain, was used to inform pain status and the interference of facial pain with patients’ activities of daily living (ADLs) during the past week, including general activity, walking, work, mood, enjoyment of life, relations with others, and sleep [19]; 2) The Patient Global Impression of Change (PGIC) [20] was used to evaluate patients’ perception of change in health compared to start of treatment; 3) The EQ-5D-5L (EuroQol-5 Dimension-5 Level) was used to evaluate health utility using the UK value set and the EQ-VAS (EuroQol Visual Analog Scale) of the EQ-5D-5L was used to evaluate global health status on the day of the survey; 4) the Brief Pain Inventory – Short Form (BPI-SF) was used to assess level of pain and general pain interference in the last 24 h [21]; 5) Depression and anxiety over the last week was assessed with the Hospital Anxiety and Depression Scale (HADS) [22]. 

### Data analysis

Descriptive analyses were used to report demographic and health information, patient-reported pain experience, treatment history and PRO instrument scores. Stratifications were made based on pain status, current medication use (monotherapy/polytherapy/no current meds), and surgery. For the surgical stratification, patients were classified as (1) “microvascular decompression (MVD) only” if having one or more MVD procedures but no non-MVD procedures for TN; (2) “Ablative surgery” if having one or more ablative procedures such as gamma knife, radiofrequency thermocoagulation, including stereotactic radiosurgery, but no MVD procedures; (3) “MVD + ablative surgery” if having procedures of both types, or (4) no surgery. Quantitative analyses were performed using SAS software (SAS Institute, Cary, USA).

## Results

### Study participants and demographics

**Fig. 1:**
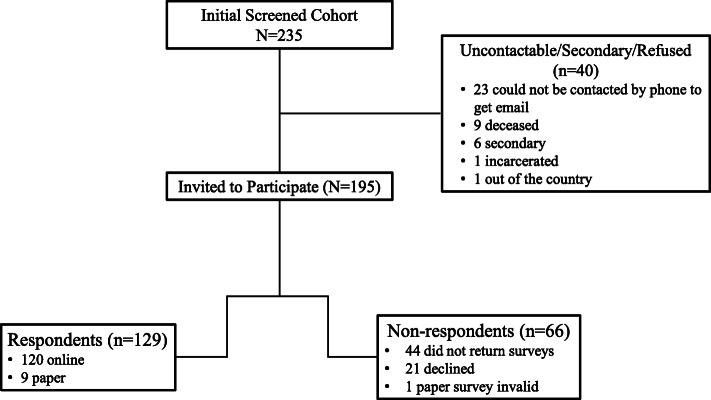
Survey Response Results

A cohort of 235 patients were initially screened for the study, of whom 195 (female 124; male 70; 1 missing) were invited to participate. Of them, 21 declined, and 174 patients (female 111; male 63) initially agreed to participate. Patients could either complete an online survey or, if they requested, were sent a paper survey. Ultimately, a total of 129 patients completed and returned usable patient surveys, which formed the basis of the analyses (Fig. 1).

Participants had been treated at this clinic for an average of over 6 years, and had an average of over 12 years since their first recorded attack.

Of the respondents, the majority were female (65%), White (88%), married (71%), ≥ 65 years (60%) and retired (56%) (Table 1). The age of respondents ranged from 39 to 85 years; the mean age was 65.7 years; median age was 67 years. Demographic characteristics are reported overall, by current pain experience, current medication use, and surgical history. There were no substantial differences between respondents and non-respondents with regards to mean age; however, among invitees, a higher percentage of those over 55 years old responded compared to younger patients. The response rate for invitees was 66% (129/195); for those who received the survey, the participation rate was 74% (129/174); 64% (111/174) were female, and of this population 76% (84/111) responded. Overall, 68% (84/124) of female invitees and 64% (45/70) of male invitees participated. The male response rate among survey recipients was 71% (45/63).

**Table 1:** TN Patient Demographics Stratified by Pain, Medication Use, and Surgical History^a^

		Pain		Medication			Surgery			
	Total Sample	Any attacks in past 4 weeks	No attacks in past 4 weeks	Current monotherapy	Current poly therapy	No current therapy	No surgery	MVD only	Ablative surgery	MVD + ablative surgery
**Total N**	129	59	69	49	35	45	77	30	13	8
**Age, Continuous (years,%)**										
Mean (SD)	65.7 (12)	66.2 (11)	65.1 (12)	69.3 (11)	64.8 (11)	62.6 (11)	67.4 (11)	61.5 (11)	69.1 (13)	60.9 (8)
Median (IQR)	67.0 (59, 73)	68.0 (58, 74)	67.0 (60, 72)	71.0 (62, 79)	66.0 (55, 74)	64.0 (57, 71)	69.0 (61, 74)	63.0 (51, 71)	71.0 (65, 82)	63.5 (53.5, 67)
Min/Max	39/85	39/85	41/85	39/85	47/85	41/79	39/85	42/79	44/84	49/70
**Age, Categories (n, %)**										
35–44	6 (5)	2 (3)	4 (6)	2 (4)	0	4 (8.9)	4 (5.2)	1 (3)	1 (8)	0
45–54	17 (13)	7 (12)	10 (15)	2 (4)	8 (23)	7 (16)	5 (7)	9 (30)	1 (8)	2 (25)
55–64	29 (23)	15 (25)	14 (20)	11 (22)	6 (17)	12 (27)	18 (23)	7 (23)	1 (8)	2 (25)
65+	77 (60)	35 (59)	41 (59)	34 (69)	21 (60)	22 (49)	50 (65)	13 (43)	10 (77)	4 (50)
**Gender, n (%)**										
Female	84 (65)	37 (63)	46 (67)	32 (65)	20 (57)	32 (71)	54 (70)	19 (63)	7 (54)	4 (50)
Male	45 (35)	22 (37)	23 (33)	17 (35)	15 (43)	13 (29)	23 (30)	11 (37)	6 (46)	4 (50)
**Race, n (%)**										
White	113 (88)	51 (86)	61 (88)	42 (86)	30 (86)	41 (91)	66 (86)	27 (90)	12 (92)	7 (88)
Asian/Asian British	8 (6)	3 (5)	5 (7)	5 (10)	1 (3)	2 (4)	7 (9)	1 (3)	0	0
Black/African/Caribbean/Black British	7 (5)	5 (9)	2 (3)	1 (2)	4 (11)	2 (4)	4 (5)	2 (7)	0	1 (13)
Other	1 (1)	0	1 (1)	1 (2)	0	0	0	0	1 (8)	0

^a^Missing *n* = 1 in some pain report and some surgery statistics

*MVD* Microvascular decompression

### Pain prevalence

Among respondents, over half (69/128; 54%) reported that they had not experienced pain in the past 4 weeks. However, only 50 patients scored a zero on the Penn-FPS-R measure, indicating no pain interference. Of the 69 who reported not experiencing pain in the past 4 weeks, 37 (54%) patients were currently not on any medication and 38 (55%) had undergone 1 or more surgical procedures (Table 2). Of those who reported pain episodes in the previous 4 weeks, the most common triggers were movement of the mouth (including talking) (35%), and ADLs (including face touching) (33%), however, just over a quarter (26%) of respondents reported spontaneous occurrence of pain.

**Table 2:** Pain Experience and Medication/Surgery – N(%)

Pain Experience in Past 4 Weeks^**a**^	Taking Medication	Not Taking Medication	Had Surgery	No Surgery
None (*N* = 69)	32 (46%)	37 (54%)	37 (54%)	31 (45%)
Yes (*n* = 59)	51 (86%)	8 (14%)	14 (24%)	45 (76%)

^a^1 participant had missing data for surgery

### Treatment history

#### Medications

All patients had taken medication for their TN pain; the most common medications taken by patients were carbamazepine and oxcarbazepine (Table 3). Other AEDs such as lamotrigine, pregabalin, and gabapentin were the next most frequently used. While some patients reported taking opioids or non-steroidal anti-inflammatory drugs during the course of their disease, these medications were not prescribed at the specialist center.

**Table 3:** History of Specific Medication Use for TN, n (%)

Medication, (***N*** = 129)	Never took	Currently taking	Took but stopped within last 6 months	Took but stopped more than 6 months ago
Carbamazepine	18 (14)	30 (23)	5 (4)	76 (59)
Oxcarbazepine^a^	43 (34)	39 (31)	9 (7)	37 (29)
Gabapentin^a^	71 (56)	8 (6)	0	49 (38)
Pregabalin^a^	92 (72)	11 (9)	2 (2)	23 (18)
Phenytoin^a^	119 (93)	2 (2)	1 (1)	6 (5)
Amitriptyline^a^	97 (76)	1 (1)	1 (1)	29 (23)
Nortriptyline^a^	119 (93)	1 (1)	1 (1)	7 (6)
Drug patches or lidocaine cream, spray^a^	111 (87)	3 (2)	2 (2)	12 (9)
Opioids, eg, morphine, fentanyl patches^a^	116 (91)	2 (2)	1 (1)	9 (7)
Tramadol^a^	114 (89)	2 (2)	2 (2)	10 (8)
NSAIDs for TN^b^	86 (68)	7 (6)	3 (2)	31 (24)
Lamotrigine^a^	94 (73)	21 (16)	6 (5)	7 (6)
Duloxetine^a^	124 (97)	2 (2)	0	2 (2)
Botulinum Toxin^a^	127 (99)	1 (1)	0	0

^a^Missing n = 1; ^b^Missing *n* = 2

*NSAIDs* non-steroidal anti-inflammatory drugs; *TN* trigeminal neuralgia

Over the course of treatment, patients had taken a mean of 3.7 medications with a range of 1–13 **(**Table 4**)**. Current mean medication use per patient was 1.1 medications, with some patients currently taking up to 5 medications. At the time of survey completion, 84/129 (65%) patients were on medication, with 49 (38%) on monotherapy and 35 (27%) on polytherapy of 2 or more medications; 45/129 (35%) were using no medications.

**Table 4:** Historical Medication Use for TN

		Current Medication Use		
	Total (*N* = 129)	Monotherapy (*N* = 49)	Polytherapy* (*N* = 35)	No Current Medication (*N* = 45)
**Number of Medications Taken for TN Ever**				
Mean (SD)	3.7 (2.2)	3.0 (1.7)	4.8 (2.5)	3.5 (2.10)
Median (IQR)	3.0 (2, 5)	3.0 (2, 4)	5.0 (3, 6)	3.0 (2, 4)
Min-Max	1–13	1–8	2–13	1–12

Nearly half of respondents (62/129; 48%) had at some point discontinued a medication due to lack of efficacy and 51/129 (40%) had discontinued due to side effects (Table 5)

**Table 5:** Reasons for Medication Discontinuation^a^

	Number of Patients, n (%)
Total Patients (N)	129
Not relieving pain	62 (48)
Changed to a different drug	59 (46)
Difficult side effects	51 (40)
No longer needed (remission)	28 (22)
No longer needed (surgery)	28 (22)
Other reason	21 (16)

^a^Patients could select more than one reason

#### Surgery

A total of 52/129 (40%) patients had undergone a surgical procedure to manage their TN. The median time since last surgery, based on 21 participants for whom these data were available, was about 3 years. The most common surgery was MVD (38/52; 73%) with 8 of these patients also having another type of surgery (Table 6). MVD was also the most recent surgery for most patients (33/52).

**Table 6:** Pain Control by Pain, Medication and Surgical History

		Pain		Medication			Surgery^**b**^			
Item	Overall	Any attacks in the past 4 weeks	No TN in the past 4 weeks	Current monotherapy	Current polytherapy	Took but Stopped	No surgery	MVD only	Ablative surgery	MVD + Ablative surgery
**Current Medication, n (%)**										
	***N*** **= 129**	***n*** **= 59**	***n*** **= 69**	***n*** **= 49**	***n*** **= 35**	***n*** **= 45**	***N*** **= 77**	***n*** **= 30**	***n*** **= 13**	***n*** **= 8**
Currently Taking Medication^a^	84 (65)	51 (40)	32 (25)	49 (38)	35 (27)	0	59 (46)	10 (8)	9 (7)	6 (5)
Not currently taking medication to manage pain	45 (35)	8 (6)	37 (29)	0	0	45 (35)	18 (14)	20 (16)	4 (3)	2 (2)
**Frequency Pattern of Pain experienced in past 6 months**^**c**^**; n (%)**										
**N**	***n*** **= 53**	***n*** **= 39**	***n*** **= 14**	***n*** **= 24**	***n*** **= 18**	***n*** **= 11**	***n*** **= 41**	***n*** **= 6**	***n*** **= 4**	***n*** **= 2**
Daily	7 (13)	6 (15)	1 (7)	4 (17)	3 (17)	NA	4 (10)	2 (33)	NA	1 (50)
For days, then I have days or weeks free of TN	17 (32)	10 (26)	7 (50)	5 (21)	6 (33)	6 (55)	13 (32)	2 (33)	2 (50)	0
For weeks, then I have days or weeks free of TN	4 (8)	4 (10)	0	3 (13)	1 (6)	NA	4 (10)	0	0	0
I have months completely free of TN	14 (26)	10 (26)	4 (29)	7 (29)	4 (22)	3 (27)	11 (27)	0	2 (50)	1 (50)
I have months free of TN attacks but with a dull ache in the background	11 (21)	9 (23)	2 (14)	5 (21)	4 (22)	2 (18)	9 (22)	2 (33)	0	0

^a^1 missing

^b^1 patient had peripheral cryotherapy before attending specialist center

^**c**^Of patients who reported pain experience in past 6 months

Overall patients had a positive post-surgical experience, with 36/52 (69%) of all patients who had surgery reporting much better pain relief including 21/52 (40%) reporting complete pain relief (Table 7). Of those who had MVD only, 17/30 (57%) reported 100% pain relief and 22/30 (73%) felt much better. However, among all patients who underwent surgery 7/52 (13%) patients felt no relief. Ten of the 52 (19%) had worsening of pain or no change in pain post-surgery. However, none of the patients reported having much worse pain after surgery and of those who had surgery, 41 (79%) stated surgery was better than staying on pain medications. When patients were asked about their most recent surgery, 15/52 (29%) of respondents reported complications, the most common of which were cerebrospinal fluid leak, unsteady on feet, and headache; other complications reported were ringing in the ears, dizziness, and facial weakness.

**Table 7:** Pain Experience by Surgical History

	Overall	MVD	Ablative surgery	MVD + Ablative surgery
	***n*** = 52^a^	***n*** = 30	***n*** = 13	***n*** = 8
**How does the current pain feel now compared to before the surgery? n (%)**				
Somewhat worse	6 (12)	4 (14)	1 (8)	1 (13)
Stayed the same	4 (8)	1 (3)	3 (23)	0
Somewhat better	6 (12)	3 (10)	0	3 (38)
Much better	36 (69)	22 (73)	9 (69)	4 (50)
0% Pain Relief	7 (13)	3 (10)	4 (31)	0
100% Pain Relief	21 (40)	17 (57)	2 (15)	2 (25)
**Currently taking any medication, n (%)**				
Currently taking monotherapy	8 (15)	2 (7)	6 (46)	0
Currently taking polytherapy	17 (33)	8 (27)	3 (23)	6 (75)

^a^1 missing: patient had peripheral cryotherapy before attending specialist center

Pain medications for TN were being taken by 25/52 (48%) who had undergone surgery (Table 6), and 17/52 (33%) reported new and different facial pain. Of the 17 patients with new and different facial pain, almost half reported recurring pain within 2 months or less of surgery (*n* = 8). Even with MVD, 5/38 (13%) required repeat MVD surgery, and all 5 of these patients still had some interference in ADLs as assessed by the Penn-FPS-R.

### 6-month pain experience

Of 53 respondents who reported pain in the past 6 months, 7 (13%) felt daily pain while 14 (26%) had pain-free periods for months. A total of 39 of these 53 participants had pain in the past 4 weeks (Table 6).

### Disease impact: PRO measures

Those with current pain interference as assessed by the PENN-FPS-R (77/127, 61%) showed numerically lower (ie, worse) quality of life scores than those reporting no pain interference on the EQ-5D-5L index (0.80, SD = 0.214 versus 0.96, SD = 0.141, respectively)​. The EQ-VAS showed similar results: those with current pain interference had lower global health scores (65.9, SD = 25.46)​ than those who did not (82.9, SD = 21.39). Patients who were not on medication and those who had undergone an MVD had numerically better overall outcomes on the PGIC and BPI measures than those who were on medication and those who did not have MVD. Patients with no current pain interference scores also reported numerically better scores on the BPI-SF pain interference scale (2.10, SD = 2.436 versus 0.18, SD = 0. 628). In the PGIC measure, which asked participants if they experienced improvement since starting treatment at this clinic, 79% expressed their condition had very much or much improved with 10 (8%) reporting worsening of their condition. The HADS anxiety and depression scores, on average, were within the normal range (0 to 7) for both those who had experienced pain episodes in the past 4 weeks and those who had not.

A summary of PRO measure results by TN attack frequency is shown in Table 8**.** A summary of PRO measure results for medication and surgical history is included in Supplement 1, Trigeminal Neuralgia PRO Scores Medication and Surgery History.

**Table 8:** PRO Scores by TN Attack Frequency

	Pain		
Measure	Overall	Any attacks in the past 4 weeks	No TN in the past 4 weeks
**Penn-FPS-R**	*n* = 127	*n* = 58	*n* = 68
Mean (SD)	22.0 (29.5)	39.1 (32.1)	7.6 (16.9)
Median (IQR)	8.0 (0.0, 36.0)	34.0 (10.0, 64.0)	0.0 (0.0, 9.5)
Min/Max	0.0/120.0	0.0/120.0	0.0/89.0
**PGIC, n (%)**	*n* = 129	*n* = 59	*n* = 69
Very much improved	63 (49)	14 (24)	48 (70)
Much improved	39 (30)	26 (44)	13 (19)
Minimally improved	11 (9)	9 (15)	2 (3)
No change	6 (5)	2 (3)	4 (6)
Minimally worse	1 (1)	0	1 (1)
Much worse	5 (4)	4 (7)	1 (1)
Very much worse	4 (3)	4 (7)	0
**BPI-SF (Intensity)**	*n* = 129	*n* = 59	*n* = 69
Mean (SD)	1.39 (1.77)	2.62 (1.89)	0.36 (0.67)
Median (IQR)	0.50 (0.0, 2.25)	2.50 (1.0, 4.3)	0.00 (0.0, 0.50)
Min/Max	0.0/7.8	0.0/7.8	0.0/2.5
**BPI-SF (Interference)**	*n* = 129	*n* = 59	*n* = 69
Mean (SD)	1.05 (1.96)	2.10 (2.44)	0.18 (0.63)
Median (IQR)	0.00 (0.00, 1.14)	1.14 (0.00, 3.86)	0.00 (0.00, 0.00)
Min/Max	0.0/9.7	0.0/9.7	0.0/3.7
**EQ-5D-5L -HUI UK weights**	*n* = 129	*n* = 59	*n* = 69
Mean (SD)	0.864 (0.2)	0.790 (0.2)	0.927 (0.2)
Median (IQR)	0.939 (0.816, 1.000)	0.841 (0.741, 0.939)	1.000 (0.924, 1.000)
Min/Max	−0.075/1.000	0.049/1.000	−0.075/1.000
**EQ-5D-5L-VAS**	*n* = 129	*n* = 59	*n* = 69
Mean (SD)	72.6 (25.2)	66.4 (24.3)	77.5 (24.9)
Median (IQR)	81.0 (55.0, 94.0)	70.0 (50.0, 88.0)	90.0 (68.0, 95.0)
Min/Max	10/100	13/100	10/100
**HADS Anxiety**	*n* = 128	*n* = 58	*n* = 69
Mean (SD)	5.4 (4.3)	6.7 (4.6)	4.3 (3.8)
Median (IQR)	5.0 (2.0, 8.0)	6.0 (4.0, 10.0)	3.0 (1.0, 6.0)
Min/Max	0.0/21.0	0.0/21.0	0.0/15.0
**HADS Depression**	*n* = 128	*n* = 58	*n* = 69
Mean (SD)	3.7 (3.8)	5.1 (5.4)	2.6 (3.0)
Median (IQR)	2.0 (1.0, 5.0)	4.0 (0.0, 9.0)	1.0 (0.0, 4.0)
Min/Max	0.0/19.0	0.0/19.0	0.0/13.0

Note: in some cases component numbers may not add up to total number due to missing data

## Discussion

This cross-sectional study, conducted at a specialist facial pain treatment facility, consisted of a subpopulation of patients of an earlier study [6]. The study yielded several key findings. Overall, the results indicate that a multidisciplinary care pathway can produce substantial benefit in a majority of patients, as evidenced by 79% of patients reporting improvement in the PGIC measure since beginning of treatment at this clinic. In addition, the average HADS score at the time of survey was within the normal range; this is encouraging in view of the fact that a prior study of 225 patients at this center (that included all 129 respondents of this study) was conducted at an earlier timepoint, and showed 36% had mild to severe depression and over 50% had anxiety as found on HADS [6]. This apparent reduction in anxiety and depression over time for the survey population is notable, given that a recent epidemiological study showed a rise in anxiety, depression and poor sleep after a diagnosis of TN [23].

Even though most patients reported improvement since beginning treatment, the majority were still on medications, with 42% of those individuals on polytherapy. At the time of the survey patients were taking an average of 1.1 medications, however overall treatment history shows patients on average had taken 3.7 drugs for TN, and as many as 13 different drugs. During the course of treatment a substantial percentage of patients had discontinued 1 or more medication(s) for various reasons, frequently due to lack of efficacy or tolerability. While polytherapy can yield clinical benefits, it also carries increased risk of AEs and drug interactions [24–26]. A study by Di Stefano et al. [9] found that carbamazepine and oxcarbazepine, the 2 medications commonly recommended as first-line therapy for TN, produce side effects that can lead to treatment withdrawal. In addition, one study reported a sizable fraction (as high as 19%) of patients experience an attenuation of the effect of carbamazepine on managing their pain over time [15], though it is not known whether this result is due to natural progression of the condition, development of resistance, or some other factor.

In this study, although surgery, especially MVD, often alleviated pain in many patients, this was not always the case: 10/52 (18%) of respondents reported somewhat worse or same pain post-surgery. Additionally, a proportion of patients required multiple surgeries and several patients experienced complications post-surgery. Further, a sizable percentage of patients continued to use medications after surgery. Also, the long-term outcome of surgery is unknown and can be deduced only from a longitudinal follow-up of this patient population. Published reports have shown that while the short-term success rate of surgery is high, there is a risk of recurrence even when initial outcomes are good, especially over time. In some cases, patients require repeat surgery or return to medications. In a follow-up study of TN patients who had MVD with a median follow-up period of 9.7 years, some patients were found to relapse even though they had immediate post-surgery relief [27]. While recurrence of pain is more likely with ablative procedures than MVD, findings from a prior study that tracked patients who had undergone MVD showed that 16.6% of them experienced a recurrence after MVD at 5 years [28]. In addition, in this survey many patients reported inadequate or no pain relief post-surgery, indicating that there are intractable cases of TN.

It should be noted that the favorable results in this study were obtained at a facility able to provide a very high level of care that consists of the concurrent involvement of healthcare practitioners from multiple disciplines, thorough monitoring of patients using established PROs, as well as prompt and thorough pharmacological and surgical interventions; this level of care may not be feasible at all conventional treatment centers [29]. Traditionally, TN has been regarded as a progressive condition that for most patients worsens over time [30, 31]. Even specialist centers not utilizing the multidisciplinary approach may fail to provide adequate care: prior to enrollment, 80% of patients who participated in the earlier study experienced inadequate disease management [6]. Even though only 40% of patients in the study population had surgery, all patients in the study were offered surgery, which in some centers may only be recommended later in the treatment pathway. Neurologists have suggested that surgical treatments should be reserved for patients with intractable TN and who have used at least 3 drugs in adequate doses [32]. As such, the favorable outcomes seen in this study may not be generalizable to all UK patients with TN.

To the authors’ knowledge, this is the first study to extensively explore the interrelated aspects of pain interference, treatment history, and patient-reported disease impact in patients who were managed following treatment recommendations of the American Academy of Neurology and the European Federation of Neurological Societies [18]. This study’s findings are supported by a study by Heinskou et al. [16], which also showed that a highly specialized center utilizing a multidisciplinary approach achieved improvement in their patients’ disease status. This approach is recommended in the updated guidelines [8]. A recent systematic review of outcomes used in 467 TN studies highlights how few studies report on domains such as physical (*n* = 46) and emotional functioning (*n* = 17) and only 35 studies collected data on patient satisfaction [33]. This study adds a breadth of information to the relatively limited prior research on these topics. Another strength of this study is that among the eligible patients initially selected for the study, no obvious difference between respondents and non-respondents was observed, indicating low risk of selection bias in the survey cohort. To further reduce bias, data collection was carried out by a third party rather than by the care providers at the treatment unit, as suggested by Akram et al. [34] Moreover, although the results reported above are cross-sectional, these data can be combined with survey data collected at an earlier timepoint from the same clinical cohort to assess longitudinal trends.

This study has some limitations. The chief limitation is that because TN is an episodic disease, the durability of pain relief is difficult to ascertain from a cross-sectional survey and it is likely to underestimate the impact of TN. Further studies with broader cohorts and data collection at multiple time points could expand upon the results presented above to account for the episodic nature of the disease and to understand the impact of TN in different patient populations. Another limitation is that because this study was conducted at a specialist multidisciplinary facility, data obtained in this study are unlikely to be applicable to the general UK TN patient population. This study also did not capture drug dosage information, and so could not assess whether those patients who had undergone surgery and were still taking medications were able to use lower doses post-surgery. Finally, the PGIC asked about change in TN since treatment began at this clinic. Given that participants had been in treatment for an average of more than 6 years, this long recall period may compromise the reliability of their responses. The response rate may have been affected by patients’ unfamiliarity with completing online surveys and the lack of suitable IT skills.

## Conclusions

These results, in conjunction with previously published results [16], indicate that a multidisciplinary care pathway is the desirable approach for all TN patients. However, it may be logistically not achievable, as conventional treatment centers may not have the resources to implement this pathway. Additionally, despite the high level of care achievable at these specialized treatment centers, there remains substantial unmet need for pain management among patients with TN. Pharmacological treatments can be effective but can have considerable drawbacks. Further, while surgery is an option when medication is not advisable or not effective, not all patients are eligible for or willing to undergo surgery and even the most effective surgery, MVD, is not successful in all cases. It is crucial that patients are aware of all available options and can change their management plans quickly depending on the status of their TN. The high numbers of current or prior medications, residual pain or complications despite surgical and/or medical interventions, and reduced QoL in those with current pain interference highlights the BOI of TN and the remaining unmet medical need. Thus, these findings support the development of additional treatment options that may result in less drug discontinuation, offer more alternatives to patients unwilling to undergo or ineligible for surgery, and be easily administered at conventional facilities. Finally, these results suggest a need for more personalization of medication use in order to obtain maximum efficacy and improved tolerability.

## Supplementary Information

**Additional file 1.** TN PRO Scores Medication and Surgery History

## Data Availability

Patients with TN at a multidisciplinary facial pain clinic within a London teaching hospital were invited to complete a survey developed by the authors. The survey included items about pain interference, history and experience of medication use, history and experience of surgery related to TN, and various PRO measures. The survey data are not publicly available. The actual questionnaire used is available upon request to the authors.

## Abbreviations

ADLs: Activities of daily living
AED: Antiepileptic drug
BOI: Burden of illness
BPI-SF: Brief Pain Inventory – Short Form
EQ-5D-5L: EuroQol-5 Dimension-5 Level
EQ-VAS: EuroQol Visual Analog Scale
HADS: Hospital Anxiety and Depression Scale
MVD: Microvascular decompression
PENN-FPS-R: Penn-Facial Pain Scale-Revised
PGIC: The Patient Global Impression of Change
QoL: Quality of life
TN: Trigeminal neuralgia
