# Supplementary material for: Burden of illness of trigeminal neuralgia among patients managed in a specialist center in England
Source: J Headache Pain. 2020 Nov 10;21(1):130. doi: 10.1186/s10194-020-01198-z (PMC7653862; doi:10.1186/s10194-020-01198-z)
Supplement: Supplementary file 1 — Additional file 1. TN PRO Scores Medication and Surgery History [file 10194_2020_1198_MOESM1_ESM.pdf]

Trigeminal Neuralgia PRO Scores Medication and Surgery History

	Medication			Surgery			
**Measure**	**Current monotherapy**	**Current polytherapy**	**Took but Stopped**	**No surgery**	**MVD only**	**Ablative surgery**	**MVD + Ablative surgery**
**Penn-FPS-R**	n=47	n=35	n=45	n=75	n=30	n=13	n=8
Mean (SD)	27.5 (28.8)	33.5 (34.3)	7.3 (18.7)	25.3 (29.4)	16.2 (25.8)	12.8 (24.1)	30.4 (46.9)
Median (IQR)	16.0 (0.0, 62.0)	17.0 (6.0, 61.0)	0.0 (0.0, 4.0)	12.0 (0.0, 56.0)	2.0 (0.0, 29.0)	0.0 (0.0, 10.0)	8.5 (0.0, 53.0)
Min/Max	0.0/83.0	0.0/120.0	0.0/94.0	0.0/101.0	0.0/98.0	0.0/76.0	0.0/120.0
**PGIC, n (%)**	n=49	n=35	n=45	n=77	n=30	n=13	n=8
Very much improved	20 (41)	8 (23)	35 (78)	35 (46)	19 (63)	7 (54)	1 (13)
Much improved	16 (33)	17 (49)	6 (13)	24 (31)	6 (20)	3 (23)	6 (75)
Minimally improved	7 (14)	2 (6)	2 (4)	10 (13)	0	1 (8)	0<br>0
No change	3 (6)	2 (6)	1 (2)	3 (4)	1 (3)	2 (15)	0
Minimally worse	0	0	1 (2)	1 (1)	0	0	0
Much worse	3 (6)	2 (6)	0	2 (3)	3 (10)	0	0
Very much worse	0	4 (11)	0	2 (3)	1 (3)	0	1 (13)
**BPI-SF (Intensity)**	n=49	n=35	n=45	n=77	n=30	n=13	n=8
Mean (SD)	1.64 (1.63)	2.06 (2.13)	0.58 (1.29)	1.63 (1.70)	0.86 (1.6)	1.29 (1.9)	1.41 (2.65)
Median (IQR)	1.00 (0.0, 3.00)	1.75 (0.25, 3.00)	0.00 (0.00, 0.25)	1.00 (0.00, 3.00)	0.00 (0.00, 1.00)	0.00 (0.0, 2.00)	0.50 (0.0, 1.25)
Min/Max	0.0/5.3	0.0/7.8	0.0/5.5	0.0/6.3	0.0/7.3	0.0/5.3	0.0/7.8
**BPI-SF (Interference)**	n=49	n=35	n=45	n=77	n=30	n=13	n=8
Mean (SD)	1.16 (1.62)	1.91 (2.76)	0.28 (1.1)	1.21 (1.96)	0.64 (1.62)	0.66 (1.45)	1.88 (3.42)
Median (IQR)	0.29 (0.00, 1.57)	0.29 (0.00, 3.71)	0.00 (0.00, 0.00)	0.00 (0.00, 1.71)	0.00 (0.00, 0.00)	0.00 (0.00, 0.29)	0.14 (0.00, 2.50)
Min/Max	0.0/5.7	0.0/9.7	0.0/6.3	0.0/9.3	0.0/6.4	0.0/4.7	0.0/9.7
**EQ-5D-5L -HUI UK weights**	n=49	n=35	n=45	n=77	n=30	n=13	n=8
Mean (SD)	0.866 (0.14)	0.789 (0.2610)	0.920 (0.1878)	0.842 (0.211)	0.916 (0.133)	0.894 (0.173)	0.823 (0.325)
Median (IQR)	0.924 (0.788, 1.000)	0.893 (0.731, 1.000)	1.000 (0.924, 1.000)	0.899 (0.792, 1.000)	0.971 (0.899, 1.000)	1.000 (0.866, 1.000)	0.950 (0.819, 1.000)
Min/Max	0.387/1.000	0.049/1.000	-0.075/1.000	-0.075/1.000	0.487/1.000	0.419/1.000	0.049/1.000

	Medication			Surgery			
**Measure**	**Current monotherapy**	**Current polytherapy**	**Took but Stopped**	**No surgery**	**MVD only**	**Ablative surgery**	**MVD + Ablative surgery**
**EQ-5D-5L-VAS**	n=49	n=35	n=45	n=77	n=30	n=13	n=8
Mean (SD)	70.6 (23.7)	68.3 (29.2)	78.1 (22.9)	69.8 (23.7)	80.2 (22.4)	76.5 (26.8)	60.5 (39.6)
Median (IQR)	76.0 (55.0, 90.0)	74.0 (51.0, 92.0)	88.0 (60.0, 95.0)	74.0 (52.0, 90.0)	87.5 (70.0, 96.0)	90.0 (50.0, 95.0)	72.0 (17.5, 96.5)
Min/Max	13/100	10/100	16/100	10/100	16/100	33/100	13/99
**HADS Anxiety**	n=48	n=35	n=45	n=76	n=30	n=13	n=8
Mean (SD)	4.8 (3.4)	7.1 (5.7)	4.6 (3.7)	5.2 (4.1)	5.6 (4.2)	4.1 (3.4)	7.9 (7.7)
Median (IQR)	4.5 (3.0, 6.0)	6.0 (1.0, 11.0)	4.0 (2.0, 7.0)	4.0 (2.5, 7.5)	5.0 (2.0, 9.0)	4.0 (1.0, 7.0)	6.0 (1.0, 14.0)
Min/Max	0.0/14.0	0.0/21.0	0.0/14.0	0.0/17.0	0.0/14.0	0.0/10.0	0.0/21.0
**HADS Depression**	n=48	n=35	n=45	n=76	n=30	n=13	n=8
Mean (SD)	3.9 (3)	5.1 (5.4)	2.4 (2.7)	4.3 (3.9)	2.4 (3.2)	2.2 (3.0)	4.8 (5.7)
Median (IQR)	3.0 (1.0, 5.5)	4.0 (0.0, 9.0)	1.0 (0.0, 4.0)	4.0 (1.0, 6.0)	1.0 (0.0, 2.0)	1.0 (0.0, 4.0)	3.5 (0.5, 6.5)
Min/Max	0.0/11.0	0.0/19.0	0.0/11.0	0.0/19.0	0.0/11.0	0.0/10.0	0.0/17.0
